# Comparison of patient satisfaction with mini-implant versus standard diameter implant overdentures: a systematic review and meta-analysis of randomized controlled trials

**DOI:** 10.1186/s40729-017-0092-4

**Published:** 2017-07-01

**Authors:** Gowri Sivaramakrishnan, Kannan Sridharan

**Affiliations:** 1 Department of Oral Health, College of Medicine, Nursing and Health Sciences, Fiji National University, Brown Street, Suva, Fiji; 2 Department of Pharmacology, Fiji National University, Extension Street, Suva, Fiji

**Keywords:** Mini-implants, Implant overdenture, Small diameter implants, Quality of life, Attachment-retained overdenture

## Abstract

Mini-implants have certain advantages over standard size implants which are being tested in various randomized controlled trials. This systematic review and meta-analysis aims to compare conventional implant overdentures to mini-implant-retained overdentures as regards to patient satisfaction. Electronic databases were searched for eligible studies data required were extracted. The extracted data were analyzed using non-Cochrane mode in RevMan 5.0 software. The heterogeneity between the studies was assessed using Forest plot, *I* ^2^ statistics, and chi-square test with a statistical *P* value of less than 0.10 to indicate statistical significance. Random-effect models were used in case of moderate heterogeneity. Four studies were included for the review and two for meta-analysis. Two studies in 177 patients comparing quality of life with mini or standard diameter implants showed a pooled result of −4.76 [−6.48, −3.04] favoring the use of mini-implants. The results for other outcomes were incomputable due to inadequate studies. GRADE approach was used for quality of life, and the strength of evidence was observed to be “low”. Mini-implant-supported overdentures had better patient satisfaction levels compared to standard diameter implant overdenture. There is definite lack of evidence to support the use of mini-implants for overdentures.

## Review

### Introduction

Implants have been considered to improve treatment outcomes of completely edentulous patients with anatomical challenges compromising the retention and stability. The root form dental implants which are 3–5 mm in diameter are considered standard diameter implants while less than 3 mm diameter implants are termed mini-implants [1]. Initially, mini-implants were used as a temporary measure, with an objective to replace it with standard diameter implants at a later date. However, they provided good stability and healing [2]. In 1997, they were approved for long-term use by the FDA. They are recently being used for complete and partial denture stabilization and also for fixed bridges [2, 3]. They are primarily indicated when there is lack of space or insufficient bone to support a standard diameter implant [3]. The survival rate of mini-implants has been reported to be 94.2% [4]. They are commonly used with ball attachment, O-rings, or a soft reline material and are usually placed using a flapless surgical procedure. Although the utility of mini-implants for implant-supported overdentures has been tested in various randomized controlled trials and case reports, a comparison between standard diameter implants and mini-implants in terms of patient satisfaction and other clinical parameters is of prime importance. Hence, the aim of this systematic review and meta-analysis is to identify patient satisfaction with mini-implant-retained overdentures compared to standard diameter implant-retained overdentures.

## Materials and methodology

### Information sources and search strategy

The protocol for this review was registered with International prospective register of systematic reviews (PROSPERO) with the registration number CRD42016043075. The review protocol can be accessed at https://www.crd.york.ac.uk/PROSPERO/display_record.asp?ID=CRD42016043075. A through literature search was conducted and was completed on 9 July 2016. The primary database used was MEDLINE (via PubMed), Cochrane Central Register of Clinical Trials (CENTRAL), and Database of Abstracts of Reviews of Effects (DARE). The search strategy was ((((((implant overdenture [tiab] OR dentur* [tiab] OR full dentur* [tiab])) AND (implan* [tiab] OR mini-implan* [tiab] OR mini implan* [tiab] OR narrow implan* [tiab] OR diameter reduced implan* [tiab])) AND Humans[Mesh])) AND ((randomized [tiab] OR randomised [tiab] OR RCT [tiab] OR clinical trial [tiab]) AND Humans[Mesh])) NOT ((review [pt] OR review [ti] OR meta-analysis [tiab] OR metaanalysis [tiab]) AND Humans[Mesh]). This search was further supplemented by hand searching of relevant references from review articles and other eligible studies. No limits were applied to the year of study but studies published only in English language were included for the present review.

### Eligibility criteria

Only those studies with randomized controlled design with the following requirements were included in the present study:

1. Type of participants—Completely edentulous patients requiring two or four mini-implants or standard diameter implants in the maxilla or mandible for implant-supported overdentures.
2. Type of intervention—Two or four mini-implants placed in the maxilla or mandible with no limits on technique of placement, loading protocol, or the attachment system used.
3. Comparison—Two or four conventional/standard diameter implants placed in the maxilla or mandible with no limits on technique of placement, loading protocol, or the attachment system used.
4. Outcome—Patient satisfaction was the primary outcome. The secondary outcomes were the outcomes measured in the included studies apart from patient satisfaction.

### Study procedure

Both the authors of this study independently screened the abovementioned databases for studies and independently reviewed abstracts for suitability. Full-text articles were obtained for those found to be eligible to be included in the review and those that were inconclusive on the abstract screening. A pre-tested data extraction form was created, and both the authors independently extracted the following data from each eligible study: trial site, year, trial methods, participants, intervention, and outcomes. Disagreement between the authors was resolved through discussion. The extracted data were analyzed using non-Cochrane mode in RevMan 5.0 software. The methodological quality of eligible trials was independently assessed by both the authors using the Cochrane collaboration’s tool for assessing the risk of bias. We followed the guidance to assess whether trials took adequate steps to reduce the risk of bias across six domains: sequence generation, allocation concealment, blinding (of participants, personnel, and outcome assessors), incomplete outcome data, selective outcome reporting, and other sources of bias. The judgment was categorized into low, high, or unclear risk of bias [5]. Percent difference between the mini-implant group (experimental) and standard diameter implant (control) was assessed from each of the eligible studies, and the mean difference in the percent and percent standard error was considered for final assessment. The heterogeneity between the studies were assessed using the Forest plot visually, *I* ^2^ statistics wherein more than 50% was considered to have moderate to severe heterogeneity, and chi-square test with a statistical *P* value of less than 0.10 to indicate statistical significance. Random-effect models were used in case of moderate heterogeneity. Considering the presence of very few trials that can be included in the review, publication bias could not be assessed. The present meta-analysis was conducted and presented in accordance with Preferred Reporting Items for Systematic Reviews and Meta-Analyses (PRISMA) guidelines [6]. The grading of quality of included studies was carried out as per Cochrane’s grading of recommendations assessment, development and evaluation tool (GRADE) [7].

## Results

### Study details

**Fig. 1:**
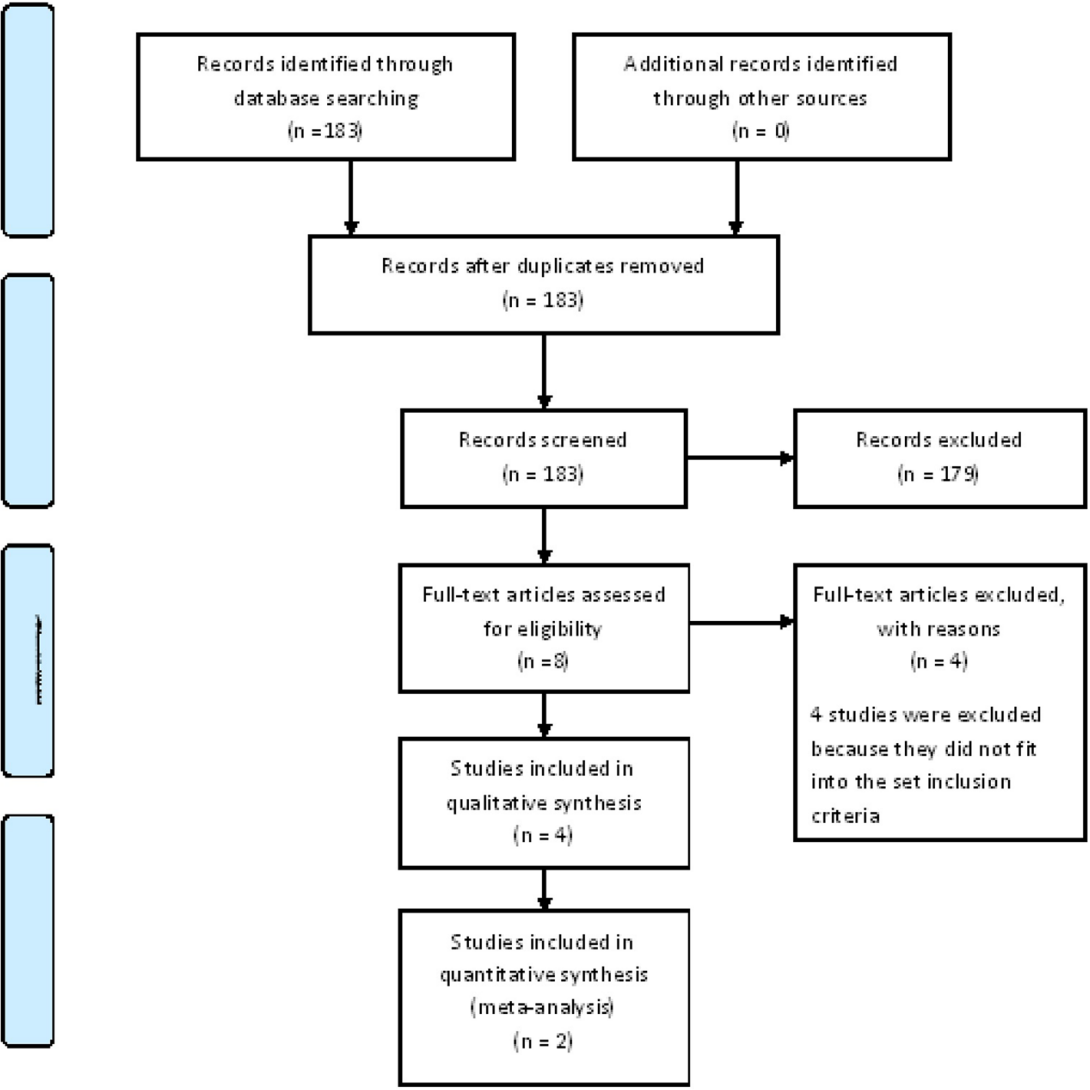
PRISMA flow diagram

**Fig. 2:**
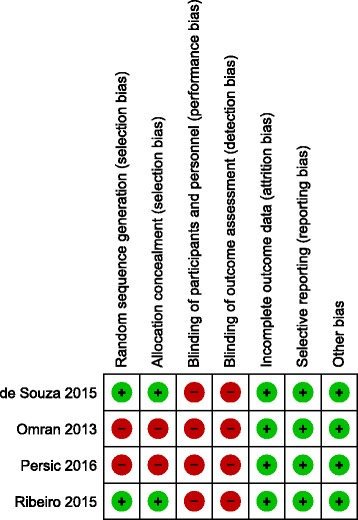
Risk of bias of the included studies

A total of 183 articles were identified using the search strategy. Screening of these papers yielded four studies comparing mini-implant-retained overdentures and standard diameter implant overdentures and were found eligible to be included in the systematic review [8–11]. Two studies [8, 11] comparing patient satisfaction between the groups were included for the meta-analysis. The PRISMA flow diagram is depicted in Fig. 1. The key characteristics of the included studies are mentioned in Table 1 [8–11]. Risk of bias of the included studies is depicted in Fig. 2.

**Table 1:** List of included studies

Author	Participant	Intervention	Comparator	Outcome
De Souza 2015[8]	120 edentulous patients completely eligible to receive implant-supported overdentures	80 patients received 2 or 4 mini-implants for implant-supported overdentures	40 patients received 2 standard diameter implants for implant-supported overdentures	Patient satisfaction as measured by oral health-related quality of life
Omran M 2013[9]	14 patients completely eligible to receive implant-supported overdentures	7 patients received 4 mini-implants for implant-supported overdentures	7 patients received 2 standard diameter implants for implant-supported overdentures	Clinical evaluation—gingival index, probing depth, radiographic evaluation
Rebeiro AB 2015[10]	120 patients completely eligible to receive implant-supported overdentures	80 patients received 2 or 4 mini-implants for implant-supported overdentures	40 patients received 2 standard diameter implants for implant-supported overdentures	VAS measured post-operative pain and discomfort
Persic S 2016 [11]	122 patients completely eligible to receive implant-supported overdentures	50 patients received 4 mini-implants for implant-supported overdentures	72 patients received 2 standard diameter implants for implant-supported overdentures	Overall patient satisfaction as measured using OHIP-14

#### Pooled results

##### Quality of life

**Fig. 3:**
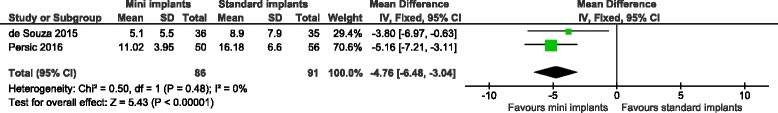
Forest plot of quality of life. A statistically significant improvement was observed in the quality of life parameter with mini implants than standard implants

Two studies [8, 11] in a total of 177 patients compared patient satisfaction with mini-implant-retained overdentures compared to standard diameter implant-supported overdentures. The pooled result was −4.76 [−6.48, −3.04] favoring the use of mini-implants (Fig. 3).

##### Other outcomes

One [9] of the included studies reported marginal bone loss, failure rate of implants, and clinical parameters and pooling of the study results was not possible indicating inadequate evidence. One study [10] reported post-operative pain and discomfort as measured by VAS and pooling of the study results was not possible due to inadequate evidence.

##### Grading the strength of evidence

GRADE approach was used for only one of the eligible outcomes which was patient satisfaction and the strength of evidence was observed to be “low” (Table 2).

**Table 2:** Grading the strength of evidence

Comparison of parameters between standard and mini-implants for implant-supported overdenture:			
Outcomes	Parameter values	No. of participants (studies)	Quality of the evidence (GRADE)
QoL	The mean QoL in the intervention groups was 4.76 lower (6.48 to 3.04 lower) than the control	177 (2 studies)	⊕⊕⊝⊝ Low^a,b^
GRADE Working Group grades of evidence High quality: Further research is very unlikely to change our confidence in the estimate of effect. Moderate quality: Further research is likely to have an important impact on our confidence in the estimate of effect and may change the estimate. Low quality: Further research is very likely to have an important impact on our confidence in the estimate of effect and is likely to change the estimate. Very low quality: We are very uncertain about the estimate.			

^a^ The total number of study population was small in both the studies

^b^ Only two eligible studies have assessed the outcome parameters

## Discussion

This study is an attempt to identify patient satisfaction with mini-implant overdentures compared to standard diameter implant-supported overdentures in completely edentulous patients. Implant-supported overdentures have been reported to offer many advantages like decreased bone resorption, reduced prosthesis movement, better esthetics, better occlusion and tooth positioning, improved occlusal load direction, and maintenance of occlusal vertical dimension. Two or four implants placed in the mandible or maxilla for implant-supported overdentures have been reported to improve quality of life compared to conventional dentures [12]. Standard diameter implants have been customarily placed; however, mini-implants have been tried in various randomized controlled trials. Mini-implants are usually less than 3 mm in diameter and are available as a single-piece system. The main advantage of using mini-implants compared to standard implants is that they could be used in individuals with large amount of bone atrophy. The other advantage of mini-implants are less invasive placement and shorter healing time, no need of bone grafts, less discomfort, and fewer complications. However, they are not indicated in patients with grinding and clenching. Four mini-implants are preferred for implant-supported overdentures in either arch [13].

Considering the advantages of mini-implants, various randomized controlled trials have been tried on mini-implant-supported overdentures for edentulous arches. Unfortunately, studies comparing mini-implants with standard diameter implants were few in number. The parameters tested in these studies were patient satisfaction, bone loss, clinical and radiographic parameters, post-operative pain and discomfort, and failures. In the present review, only four studies were identified comparing standard diameter implants to mini-implants for overdentures in edentulous patients. Only two out of the four reported patient satisfaction and this was found to favor mini-implants. The other outcomes measured in these included studies could not be pooled because of lack of sufficient data. This indicates a definite lack of evidence to compare mini-implants to standard diameter implants for overdentures. Considering the advantages of mini-implants, more high-quality randomized controlled trials comparing mini with standard diameter implants are to be initiated. These trials should be based on testing both patient satisfaction and also other clinical and radiographic outcomes measuring overall success of these implants for implant-retained overdentures.

## Conclusion

However, considering the results obtained from available evidence, mini-implants tend to provide good patient satisfaction compared to standard diameter implants when used for implant-supported overdentures. The results of this meta-analysis should be interpreted keeping in the mind the limited availability of data to be included. This paper would serve as a basis for future research comparing mini-implants with standard diameter implants for implant-supported overdentures.
